# Coexpression analysis identifies nuclear reprogramming barriers of somatic cell nuclear transfer embryos

**DOI:** 10.18632/oncotarget.19504

**Published:** 2017-07-22

**Authors:** Yongchun Zuo, Guanghua Su, Lei Cheng, Kun Liu, Yu Feng, Zhuying Wei, Chunling Bai, Guifang Cao, Guangpeng Li

**Affiliations:** ^1^ The Research Center for Laboratory Animal Science, College of Life Sciences, Inner Mongolia University, Hohhot 010021, China; ^2^ College of Veterinary Medicine, Inner Mongolia Agricultural University, Hohhot 010018, China

**Keywords:** somatic cell nuclear transfer (SCNT), gene co-expression analysis, enrichment of GO category, pathway aberrant activation, reprogramming barriers

## Abstract

The success of cloned animal “Dolly Sheep” demonstrated the somatic cell nuclear transfer (SCNT) technique holds huge potentials for mammalian asexual reproduction. However, the extremely poor development of SCNT embryos indicates their molecular mechanism remain largely unexplored. Deciphering the spatiotemporal patterns of gene expression in SCNT embryos is a crucial step toward understanding the mechanisms associated with nuclear reprogramming. In this study, a valuable transcriptome recourse of SCNT embryos was firstly established, which derived from different inter-/intra donor cells. The gene co-expression analysis identified 26 cell-specific modules, and a series of regulatory pathways related to reprogramming barriers were further enriched. Compared to the intra-SCNT embryos, the inter-SCNT embryos underwent only complete partially reprogramming. As master genome trigger genes, the transcripts related to TFIID subunit, RNA polymerase and mediators were incomplete activated in inter-SCNT embryos. The inter-SCNT embryos only wasted the stored maternal mRNA of master regulators, but failed to activate their self-sustained pathway of RNA polymerases. The KDM family of epigenetic regulator also seriously delayed in inter-SCNT embryo reprogramming process. Our study provided new insight into understanding of the mechanisms of nuclear reprogramming.

## INTRODUCTION

Somatic cell nuclear transfer (SCNT) is a technology to create an exact genetic match of the donor by transferring the donor nucleus into the enucleated recipient oocyte [1]. SCNT has immense potential to generate patient-specific pluripotent stem cells for regenerative medicine and specific therapies [2]. The recent high-profile study reported that the ES cells reprogrammed by SCNT showed more similar epigenetic and transcriptional signatures remarkably to those of embryos produced based on *in vitro* fertilization [3]. However, the efficiency is extremely low and most cloned embryos usually arrest at early development, the mechanisms that underlie the cell nuclear reprogramming remain poorly understood [4].

It is generally believed that the principal cause of developmental abnormalities of SCNT embryos is aberrant nuclear reprogramming of the donor somatic cells [5]. Compared to the fertilized preimplantation embryos, the embryos derived from SCNT had the added challenge of silencing of donor nuclear transcriptions while reactivating all of the embryo-related genes [6]. Upon transfer of a somatic nucleus to an enucleated recipient oocyte during the cloning process, several essential changes must ensue [7, 8]. In doing so, it must also shed its differentiated phenotype and gain a new pluripotent state. All these changes involve a remodeling, not of the underlying genetic sequences that comprise the genome, but of the epigenetic regulator also play crucial roles reestablishment of well-orchestrated gene expression [9, 10]. In this process, we believe that there are many barriers in the cell reprogramming process and the underlying molecule mechanisms are poorly unraveled.

Interspecies SCNT (inter-SCNT) is defined as the procedure by which somatic nuclei introduced into the oocyte’s cytosol of a different species, presents a larger biological challenge [11]. The inter-SCNT is an ideal way for revealing the nuclear-cytoplasmic interactions, generating autologous ESCs and cloning endangered animal species [12]. It provides an extreme case of reprogramming failures from which much can be understood regarding the basic biological mechanisms underlying genome reprogramming [13–15].

All reprogramming events critically depend on a controlled and orchestrated program of gene expression [3, 16, 17]. Deciphering the temporal and spatial patterns of gene expressions in both intra- and inter-SCNT embryos are crucial step toward understanding the mechanisms of nuclear reprogramming. In order to investigate the mechanisms involved in the SCNT reprogramming, we collected more than 2000 cloned embryos from four different inter-family donor cells, established valuable transcriptome recourse of SCNT embryos. Based on weighted gene co-expression network (WGCNA) approach, the cell-specific modules were identified, and those module significance and GO enriched categories were analyzed. Then, we compared the regulatory pathways of reprogramming barriers by GO category analysis. At last, the molecular mechanism that caused the developmental failure of inter-SCNT cloned embryos was further discussed.

## RESULTS

### Total gene expression profiles of SCNT embryos derived from different species

**Figure 1:**
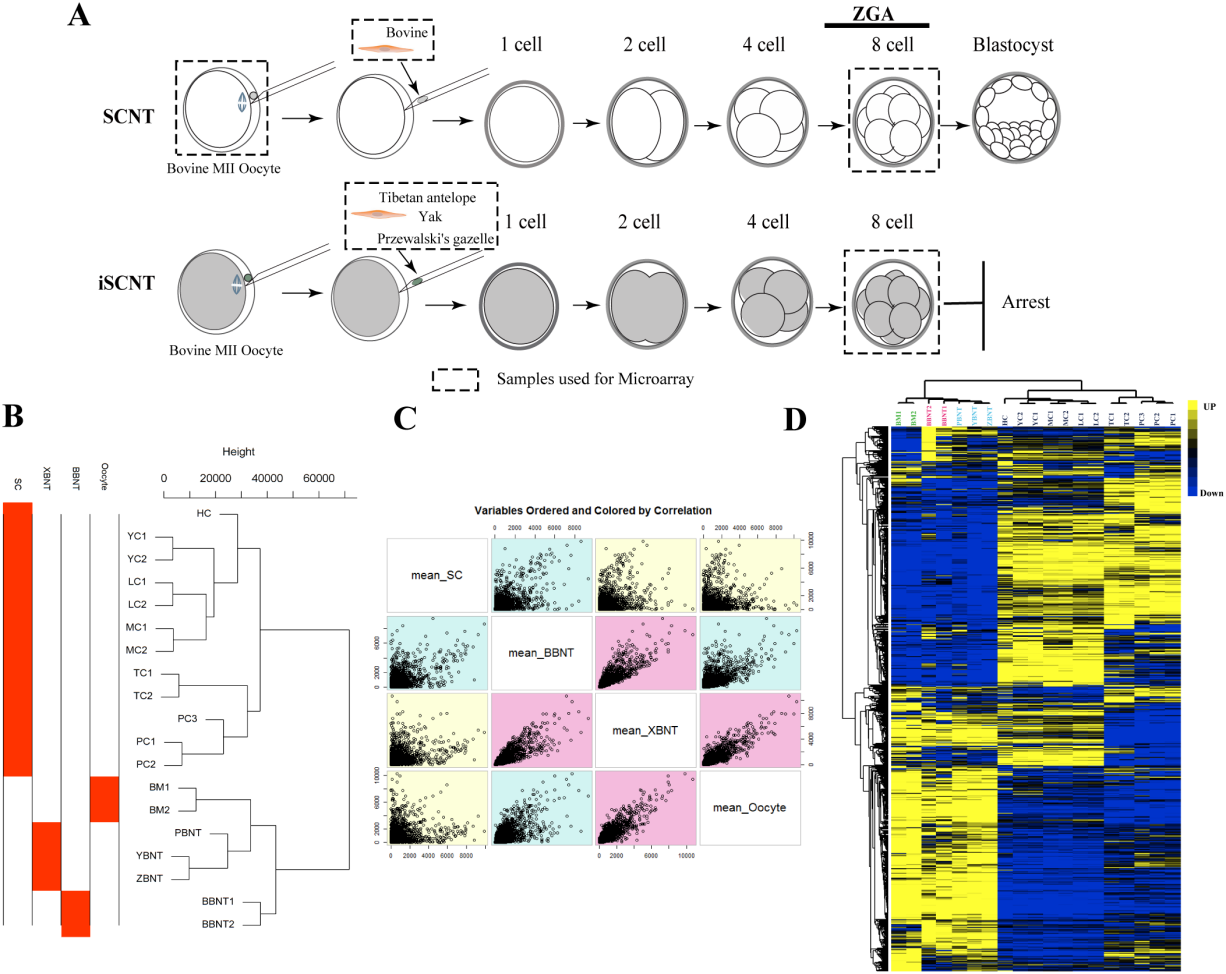
Schematic illustration for exploring reprogramming barriers and overview of total gene expression variation **(A)** Schematic illustration of the experimental procedures and cell sample collections. Samples used for microarray are marked by dashed rectangles. **(B)** Unsupervised clustering of global genes. **(C)** Variable ordered of sample pairs and colored by correlation. **(D)** Heatmap comparing transcription levels of the global transcriptome profile of different samples. Rows represent genes and columns represent samples. For a gene, yellow represents the higher expression level, blue represents the lower expression level and black represents the medial expression level for all samples.

The inter-SCNT is an ideal method for studying the nuclear-cytoplasmic interactions of cell reprogramming. From the *in vitro* development experiment of embryo listed in Table 1, we can observed that the blastocyst development efficiency of inter- SCNT (include TBNT, PBNT, YBNT, also called XBNT) is significantly lower than that of intra-SCNT (also called BBNT). The 8-16 cell stage is the most critical period for early embryo development. The embryonic genome activation (EGA) is crucial for the beginning of self-sustained cellular biology, which takes place at 8-16 cell stage in bovine embryos [18]. To identify the earliest transcriptional differences between 8-cell embryos derived through inter-SCNT and intra-SCNT, we performed microarray experiments using pooled embryos (450-500 embryos/samples) at 8-cell stages (Figure 1A). A valuable transcriptome recourse of SCNT embryos was established, which derived from more than 2000 cloned embryos from four different inter-family donor cells (Table 2). Sample-by-sample correlation matrix was calculated and unsupervised hierarchal clustering dendrogram showed that 19 samples are accurately clustered into four distinct classes (Figure 1B). We observed that most of the replicates clustered together and the variation of intra-samples were smaller than inter-samples (Supplementary Figure 1).

**Table 1:** The development rate of *in vitro* cloning embryo

Intra/inter	No. embryos cultured	Cleavage (%)	8-16 cells (%)	Blastocysts (%)
TBNT	375	303 (80.8%)	266 (70.9%)	3 (0.8%)
PBNT	279	221(79.2%)	193(69.1%)	3(1.1%)
YBNT	356	270(75.8%)	220(61.8%)	60(16.8%)
BBNT-1	256	203(79.3%)	108(70.3%)	54(21.1%)
BBNT-2	388	318(82.0%)	279(71.9%)	89(22.9%)

**Table 2:** Summary of primary samples used in this study

ID	Full name	Replication	Number of pooled embryos
1	Bovine metaphase II stage oocytes (BM)	2	1050
2	Bovine metaphase II stage oocytes (BM)		1000
3	Bovine–bovine somatic cell nuclear transfer embryos (BBNT)	2	309
4	Bovine–bovine somatic cell nuclear transfer embryos (BBNT)		552
5	Przewalski’s gazelle–bovine nuclear transfer embryos (PBNT)		527
6	Yak–bovine nuclear transfer embryos (YBNT)	3	521
7	Tibetan–bovine nuclear transfer embryos (TBNT)		515
8	Luxi cattle somatic cells (LC)	15	-
9	Luxi cattle somatic cells (LC)		
10	Mongolia cattle somatic cells (MC)		
11	Mongolia cattle somatic cells (MC)		
12	Holstein somatic cells (HC)		
13	Yak somatic cells (YC)		
14	Yak somatic cells (YC)		
15	Tibetan somatic cells (TC)		
16	Tibetan somatic cells (TC)		
17	Przewalski’s gazelle somatic cells (PC)		
18	Przewalski’s gazelle somatic cells (PC)		
19	Przewalski’s gazelle somatic cells (PC)		

The scatter plot of variable orders of sample pairs and correlation color identified three distinct segmentations, oocyte, embryo and somatic cells (Figure 1C). For whole gene expression pattern, the transcriptome profiles across different cell types showed the oocyte and embryos were with the consistent expression patterns (Figure 1D). Compared to inter-SCNT (XBNT) embryos, more EGA transcripts were upregulated in intra-SCNT (BBNT) embryos at 8-cell stage (Figure 1D). This indicated that the BBNT embryos occur maternal-zygotic transition more comprehensive than XBNT during early embryogenesis.

### Global different gene expression at the time of EGA

**Figure 2:**
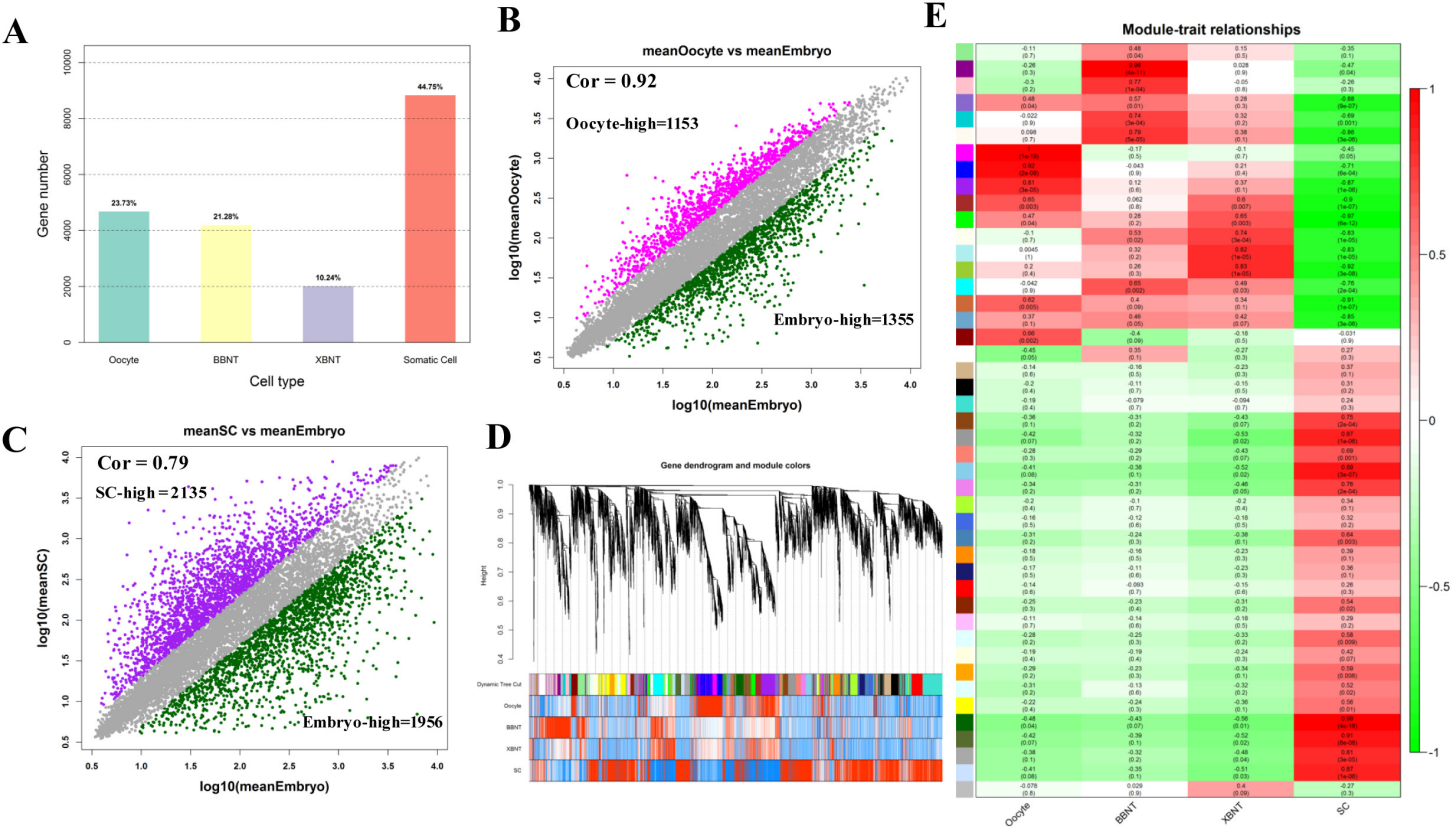
The global landscape of differentially expressed genes and gene coexpression analysis of cell-specific dynamics transcriptiomes **(A)** The proportion of the highest activated transcripts for different cell types. **(B** and **C)** Scatter plot compares the differentially expressed gene distribution pattern for oocyte, Embryo and Somatic cells, Spearman correlation are using in this calculated. **(D)** Clustering dendrogram obtained with the weighted correlation network analysis. The first color row underneath (labeled group) shows the module assignment determined by the Dynamic Tree Cut. The other color rows represent the module location of different development stages. **(E)** Cell specific co-expression gene modules and their correlation to development stage based on WGCNA analysis. Numbers of each square represent correlation of module and development stage, and p-value of each correlation value. Color of each square is correspond to correlation: Positive correlation (Red); Negative correlation (Green); No correlation (White).

It is evident that the major barrier that hinders the developing SCNT embryos are mainly appeared at the EGA stage. The bar graphs in Figure 2A showed the proportions of transcripts with max value of expression level in different cell lines. The proportions is increased from min for XBNT embryos (2022 transcripts, 10.24%) to max for somatic cells (8840 transcripts, 44.75%) (Supplementary Figure 2). When adding up the embryonic development associated genes, we can observed the development transcripts and the somatic cells transcripts each half of the totally transcripts. This indicated that about half of coding genomic regions will be reactivated during reprogramming process of somatic cell. The transcriptome of oocyte and embryos showed high identity (spearman correlation (cor) =0.92) (Figure 2B), about 2000 differentially expressed genes were identified. Transcriptome comparison between somatic cells (SC) and embryos at the 8-cell stage identified 4091 genes, demonstrated more expression difference existed between somatic cells and embryos (Figure 2C).

**Figure 3:**
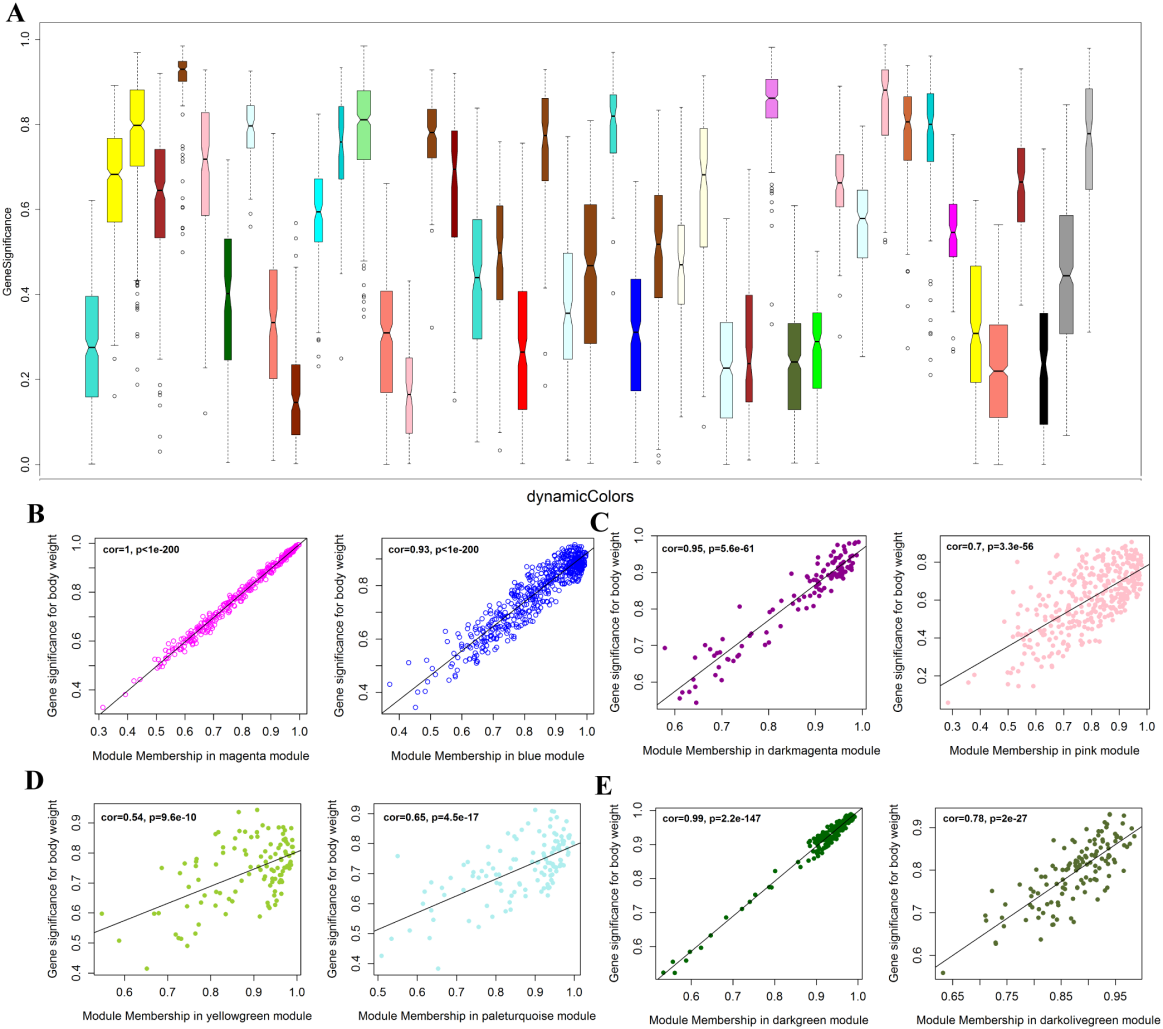
Module significance of cell specific co-expression genes and their correlation to developmental stages **(A)** Boxplot of gene significance for 47 modules of coexpressed transcripts. **(B, C, D, F)** Scatter relationship between module membership and gene significance. (B) magenta and blue modules are significant to oocytes; (C) dark magenta and pink modules are significant to BBNT embryo; (D) yellow green and pale turquoise modules are significant to XBNT; **(E)** dark green and dark olive green modules are significant to Somatic Cells (SC).

To comprehensively characterize the transcriptome difference between different cell types, the weighted gene co-expression network analysis (WGCNA) was further performed for module analysis of co-expression. Figure 2D showed hierarchical clustering dendrogram for the co-activation pattern of whole genome. The profiles were clearly clustered into three clear patterns, representing unique signature of oocyte, embryo and somatic cell, respectively. Total of 45 modules were identified by using co-expression analysis (Figure 2E, Figure 3A, Supplementary Table 1 and Supplementary Figure 3). And 26 modules have significant cell specificity (cor >0.6, p<0.05) (Supplementary Table 2 and Supplementary Table 3), 6 modules preferred to oocytes (Figure 2E, Figure 3B), 5 modules preferred to BBNT embryos (Figure 2E, Figure 3C), 5 modules preferred to XBNT embryos (Figure 2E, Figure 3D), and 10 modules preferred to somatic cells (Figure 2E, Figure 3E). The expression patterns of these modules were well-differentiated for cell types.

### Module significance and cell specific selection

In order to identify co-expression modules, we further analyzed the module significance and their correlation with cell types. The darkgreen module had the highest gene significance, showed the gene cluster was the nearest to the somatic cells. We can found the expression patterns of these modules were well-differentiated among development stages. In addition, we also analyzed the gene significance of every module to verify the correlation between the identified module and cell type (Figure 3B-3E, Supplementary Figure 4).

**Figure 4:**
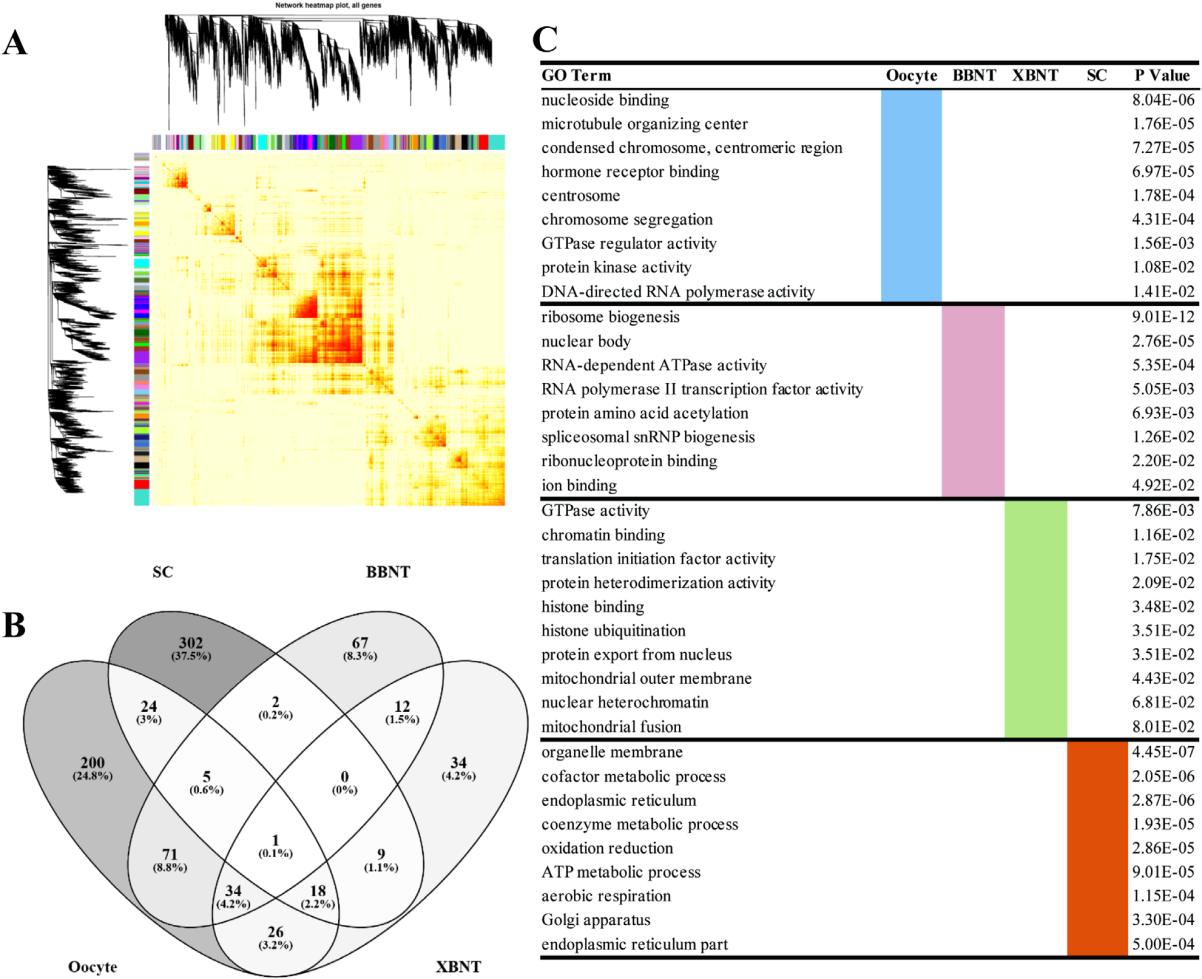
The heatmap plot of gene network and the GO enrichments of differentially expressed genes **(A)** The Topological Overlap Matrix (TOM) heatmap of epigene network. Light color represents low overlap and progressively darker red color represents higher overlap. Blocks of darker colors along the diagonal are the modules. The gene dendrogram and module assignment are also shown along the left side and the top. **(B)** Venn diagram of shared and unique GO terms among different transcriptomes. **(C)** The cell-specific GO enriching categories for differentially expressed genes.

The magenta module showed the largest correlation between module membership and gene significance (cor = 1.0, p=1.e-200) (Supplementary Figure 3). Thus, we can conclude that the magenta module plays the most important role in oocyte, then the blue (Figure 3B, Supplementary Figure 5). The dark magenta module is specific to BBNT cell, then the pink (cor=0.95, p=5.6e-61, Figure 3C, Supplementary Figure 6) and the dark green module is specific to somatic cells, then the light steel blue (cor=0.99, p=2.2e-147, Figure 3E, Supplementary Figure 7). The lowest correlation was the XBNT magenta module. The highest cor value was only up to 0.65, however it is still significant specific to interspecies SCNT cells (p=4.5e-17, Figure 3D, Supplementary Figure 8). Based on the correction coefficient analysis, the specific modules of cell types can be clustered separately (Figure 3). To provide deeper insights into the transcriptomic diversity of cellular processes, we constructed a coexpression network and analyzed its topological properties. The heatmap showed that genes within modules display more topological overlap than the across modules (Figure 4A).

### Functionality analysis of GO enriched categories for differentially expressed genes (DEGs)

To gain more insight into the EGA difference between different cell types, the cell specific transcripts have been clustered based on WGCNA. When using cor=0.74 as cutoff, three oocyte-specific modules, four BBNT-specific modules, three XBNT-specific modules and eight SC-specific modules were identified for further analysis of GO functional enrichment (Supplementary Table 4). There were 1257 non redundant DEGs consistent with oocyte-specific expression were enriched into 379 non-repeated GO categories (Figure 4B and 4C, Supplementary Table 5). Two hundred of 379 (24.8%) are belonged to oocyte-specific modules (Supplementary Table 6), genes were mainly involved in many activity and chromatin organization biological processes such as nucleoside binding, chromosome segregation, protein kinase activity, and DNA-directed RNA polymerase activity (Figure 4C).

For cloned embryos specific modules, 711 and 328 non redundant DEGs were identified for BBNT(intra-SCNT) and XBNT(inter-SCNT) embryos, 192 and 134 non-repeated GO categories were enriched, respectively (Figure 4B, Supplementary Table 5). And 67 categories included exclusively in BBNT embryos and 34 categories were belonged to XBNT embryos (Supplementary Table 6). The numbers of BBNT embryos are two times than those in XBNT for both DEGs and GO categories. This indicates that the intra-SCNT embryo successfully activated more widely reprograming-related genes compared to inter-SCNT embryo. This ensures the BBNT embryo gain higher efficiency of blastocyst. For 12 common categories in BBNT and XBNT, All of them belong to housekeeping pathways of development. For example, the general embryo-specific biological pathways were identified, which contained ribosome biogenesis, amino acid acetylation, histone modification, mitochondrial fusion, translation initiation factor activity and so on (Figure 4C). For somatic cells, 1165 genes of eight SC-specific modules were observed, 302 unique categories were enriched into the basic cell metabolic events, including organelle membrane, endoplasmic reticulum, oxidation reduction, ATP metabolic process, Golgi apparatus, and endoplasmic reticulum part (Figure 4C, Supplementary Table 5, and Supplementary Table 6). Venn diagram showed more common categories were shared by embryos and oocytes.

### Identification of reprogramming barriers in SCNT embryos

**Figure 5:**
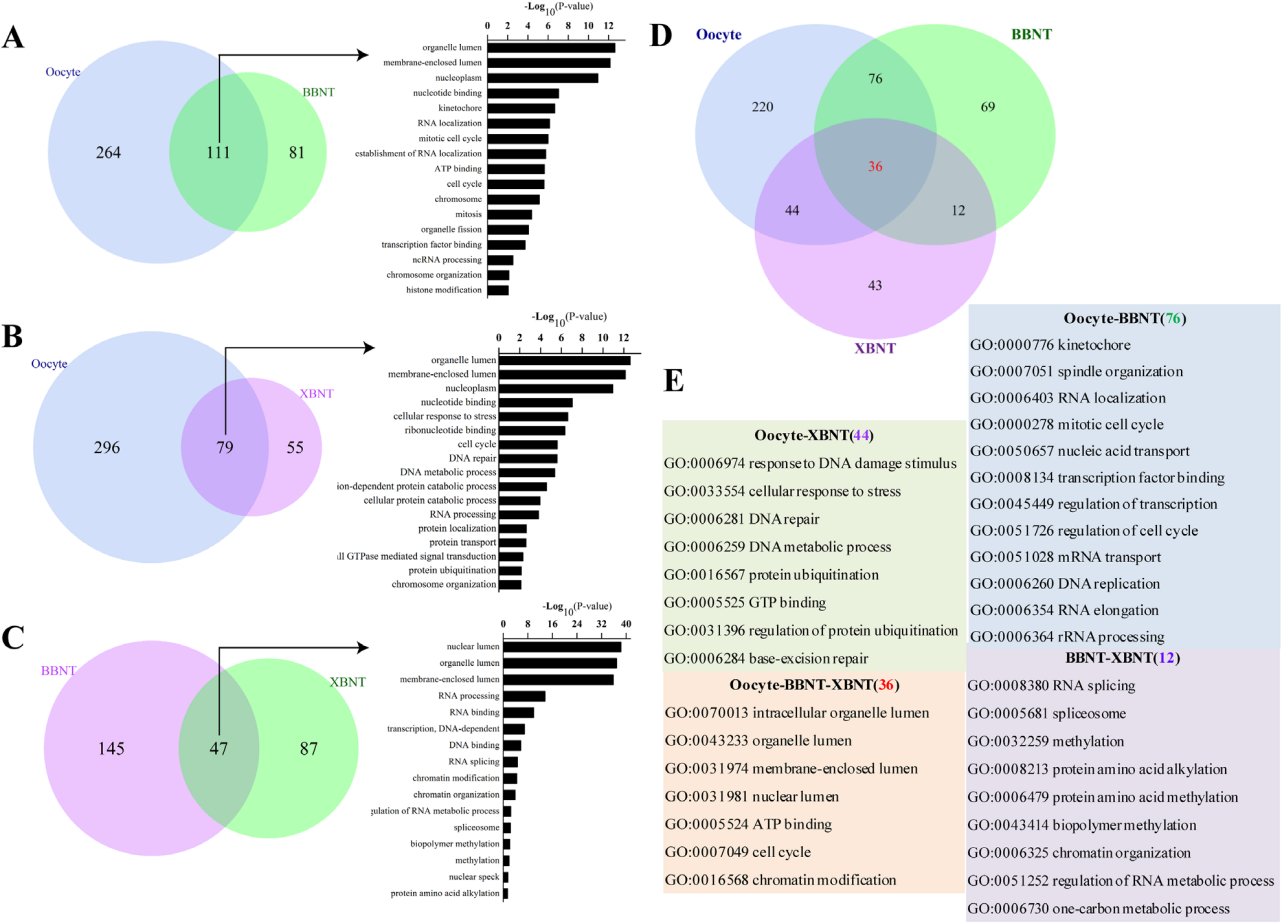
The overlap of GO terms and functional classification categories of paired embryos Venn diagram showing the shared and unique GO terms for oocyte vs. BBNT **(A)**, oocyte vs. XBNT **(B)** andBBNT vs. BBNT **(C)**. **(D)** Venn diagram showing the unique shared GO terms for oocyte, XBNT and BBNT. **(E)** Gene ontology categories of unique shared between any two embryo types classified in (D).76GO terms exclusively included in both oocyte and BBNT, 44GO terms exclusively included in both oocyte and XBNT, 12GO terms exclusively included in both XBNT and BBNT, oocyte, BBNT and XBNT shared 36GO terms.

To explore the regulatory pathways of reprogramming barriers in SCNT embryos, pairwise comparison of GO categories for oocytes, 2-cell IVF, and 2-cell SCNT embryos were analyzed. There were 111 common categories were shared in both oocyte and BBNT (Figure 5A), 79 common categories were shared in oocyte and XBNT (Figure 5B), and 47 common categories were shared in BBNT and XBNT (Figure 5C). These 36 common GO categories shared in three cell types, which were involved coherent biological effect: membrane-enclosed lumen, RNA processing, RNA biosynthetic process and chromosome organization (Figure 5D and 5E). This study gave consistent results to a latest published single-cell RNA sequencing analysis which identified three pathways in human 8-cell stage embryos [19, 20].

The common categories in oocyte and BBNT were preferred embryo normally activity of mRNA and proteins stored in the oocyte cytoplasm, such as spindle organization, cell cycle and RNA processing. In the XBNT, the situation was even more complicated because the donor nucleus and recipient cytoplasm originated from different species. The common categories in oocyte and XBNT demonstrated that the DNA damage stimulus (GO:0006974), cellular response to stress (GO:0033554), DNA repair (GO:0006281), protein ubiquitination (GO:0016567), and base-excision repair (GO:0006284) were most urgent molecular events in the heterogenous embryos. Meanwhile, the XBNT cells and BBNT cells also showed coherent biological effect of EGA, including RNA splicing, spliceosome, methylation, alkylation, chromatin organization.

### The aberrant activation of pioneer master regulator in both cloning embryos

**Figure 6:**
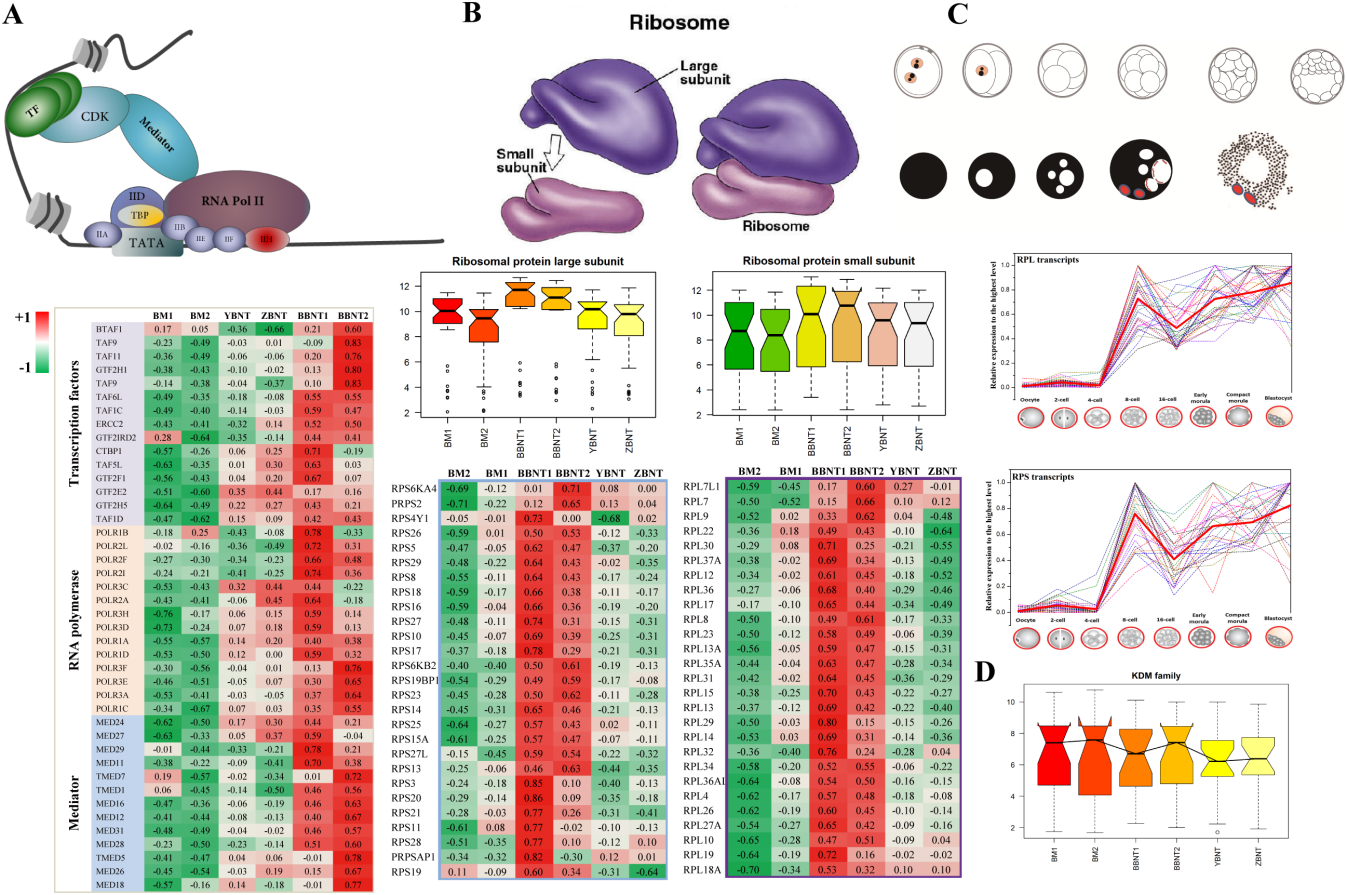
Heatmap of abnormal activation of key functional pathways in maternal-zygotic transition event **(A** and **B)** The abnormal expression of pioneer regulator for recruiting transcriptional machinery (A) and assembling ribosome subunit (B). **(C)** Relative expression of ribosome related transcripts during mammalian preimplantation embryogenesis (Data from Jiang et al. BMC Genomics. 2014 15:756). **(D)** Barplot of KDM family for different cell types.

Large scale synthesis of mRNA from the diploid embryonic genome is initiated at a species-specific time point [21]. This occurs at the 8-cell stage in bovine and human embryos [22, 23]. To further insight into the difference of EGA between intra-SCNT and inter-SCNT cloned embryos, we re-filtered the identified GO categories of each module. Several pioneer regulatory pathways ware clearly aberrant activation in XBNT embryos. Significant differences between the mRNA expression profiles were observed in pathway of transcription regulation [24]. All the three families of transcriptional regulation demonstrated that incomplete reprogramming of donor cells occurred in the yak-bovine and Tibetan-bovine SCNT embryos (Figure 6A, Supplementary Table 7). For the basal transcription factors, both oocyte and embryos contained higher transcript activation than the somatic cells (Supplementary Figure 9). For the RNA polymerase, however, the highest activation was only occurred in BBNT cells. This may be the transcripts of basal transcription factors had already stored in oocyte cytoplasm, which belonged to the maternal family [25]. The RNA polymerase are embryonic genome pathway, the BBNT embryos successfully activated their self-sustained genome. But the XBNT failed activated because of the incompatibility between donor nuclear and receptor cytoplasm.

It is well known that ribosome assembly plays the most important roles in EGA at the 8-cell stage (Figure 6B). However, the co-expression comparison between intra-SCNT and inter-SCNT embryos showed that both the large and small subunits of ribosome were assembled failure during early communication between nucleus and cytoplasm (Figure 6B and 6C). For the epigenetic reprogramming of SCNT embryos, we also confirmed that the KDM family was also inactivated in the XBNT embryos (Figure 6D).

For the SCNT, the somatic cell nuclei must undergo extensive reprogramming for successful development of the cloned embryos [26]. However, it was recently demonstrated that the somatic cell nucleus undergoes only partial or incomplete reprogramming in inter-SCNT embryos [27]. The present results of co-expressed analysis were further indicated that a significant number of DGEs were activated between the BBNT and XBNT embryos at 8-16 cell stage. Many key reprogramming regulatory pathways were significantly down regulated in 8-16 XBNT embryos [28].

## DISCUSSION

SCNT has been successfully utilized in the production of many mammalian species including laboratory and domestic animals [29, 30]. However, extremely poor development rate of SCNT embryos limited its extensive huge potentials [31, 32]. A successful SCNT procedure depends on multiple factors, such as oocyte quality, enucleation, cell fusion, activation, culture medium, and cell cycle stage of the donor nuclei [33]. Lots of previous studies have reported that incomplete genomic reprogramming may be the major barrier of cloned embryos development [34, 35]. During nuclear reprogramming, a precise and accurate communication between nucleus and cytoplasm determines the cloning success.

Because the donor nucleus and recipient cytoplasm are originated from different species, the inter-SCNT is desired model for nuclear reprogramming research and a powerful tool for discovering the master genome activation genes [13, 14]. To identify the earliest transcriptional differences between 8-cell embryos derived through both inter-SCNT (XBNT) and intra-SCNT (BBNT), we performed microarray experiments using pooled embryos (450–500 embryos/samples) at 8-cell stages. The first valuable transcriptome recourse of SCNT embryos we established, which derived from four different inter-family donor cells, and sample-by-sample correlation matrix was calculated. Unsupervised hierarchal clustering dendrogram showed the efficiency of inter-SCNT is inversely proportional to the evolutionary distance among the species. The variation of inter-samples tends to be smaller than intra-samples. The global different gene expression based on transcriptome comparison demonstrated that inter-SCNT must undergo more complicated nucleus-cytoplasm communication for accomplishing the EGA [5, 36]. Compared to the BBNT embryos, the XBNT embryos experienced only partially incomplete reprogramming at 8-cell stage. The mitochondrial heteroplasmy may not be a major cause of developmental failure in cytoplasmic hybrid embryos [6, 37].

Based on WGCNA approach [38, 39], we provided a deep transcriptome analysis of DEGs for SCNT embryo. Totally of 45 modules were identified, in which 26 modules were performed significant cell specificity. The expression patterns of these modules were well-differentiated among development stages. The coexpression network and topological properties showed more topological overlap than the genes across modules according to the topological overlap heatmap in the gene network. The further GO categories comparison identified 200 oocyte-specific modules, 67 categories BBNT-specific modules, 34 XBNT-specific modules and 302 SC-specific modules. Their functional enrichment can well reflect the cell-specific pathway marker. The aberrant activation of master regulators in intra-SCNT and inter-SCNT embryos demonstrated that the pioneer factors, present in the oocyte cytoplasm, were failed to bind the sequence target on the heterology nuclear genome from another species [6, 24].

It is well known that maternal pioneer sequence-specific transcription factors play critical role for opening ZGA [40, 41]. As master genome trigger genes, the transcripts related to TFIID subunit, RNA polymerase and mediator were incomplete activated in inter-SCNT embryos. If cloning embryos that fail to accomplish this task, they do not survive beyond the eight-cell stage. The different expression results of basal transcription factors and RNA polymerase confirmed our conclusion. The XBNT only wasted the stored maternal mRNAs, but failed to activate their self-sustained cellular biology. The genomic incompatibility between the nuclear donor cell and the cytoplast may be as a major contributing factor causes the developmental failure of inter-SCNT cloned embryos [5, 6, 12, 37]. Finally, one of epigenetic decisive factors, KDM family, was further analyzed. The result was consistent with the latest high-profile studies [16, 28]. Thus, we speculate that the uncompleted activation of transcription and epigenetic reprogramming may reduce inter-SCNT embryo genome activation and resulted in extremely poor embryo development.

## MATERIALS AND METHODS

### Ethics statement

All of the bovine oocytes and embryos were handled according to the guidelines of The Inner Mongolia University Animal Care and Use Committee. The bovine ovaries used in this study were collected with permission of the Hohhot slaughterhouse. The animal protocol was approved by The Animal Care and Use Committee of Inner Mongolia University. The small pieces of ear tissue of an adult Przewalski's gazelle and Tibetan antelope were collected in Qinghai Wildlife Garden (Xining, Gansu) with the permission of Qinghai Forestry Bureau. The Mongolia cattle somatic cells (MC) were collected from the Alax Banner of Inner Mongolia; The Holstein somatic cells (HC) were collected from Hohhot, Inner Mongolia; The Yak somatic cells (YC) were collected from Gansu province; The Luxi cattle somatic cells (LC) were collected from Shandong province.

### Data collection

In embryos group, we collected two biological replicates containing 1050 oocytes (BMII, ID-1) and 1000 occytes (BMII, ID-2) respectively; two biological replicates of bovine-bovine intraspecies cloned embryos (BBNT), containing 309 (ID-3) and 552 (ID-4) BBNT embryos; 527 (ID-5) Przewalski's gazelle-Bovine interspecies nuclear transfer 8- to 16-cell stage embryos (PBNT), 521 (ID-6) Yak-Bovine interspecies nuclear transfer 8- to 16-cell stage embryos, 515 (ID-7) Tibetan-bovine interspecies nuclear transfer 8- to 16-cell stage embryos (TBNT). In somatic cell group, we collected two biological replicates Luxi cattle somatic cells (LC, ID- 8, 9), Mongolia cattle somatic cells (MC, ID-10, 11), Yak somatic cells (YC, ID-13, 14) and Tibetan somatic cells (TC, ID-15, 16) respectively. In addition, we also collected Holstein somatic cells (HC, ID-12) and three replicates Przewalski’s gazelle somatic cells (PC, ID-17, 18, 19). The summary of collected samples used in this study was listed in Table 2.

### Transcription profiling

Total RNA of primary 19 samples was extracted, processed and hybridized to the Affymetrix GeneChip Bovine Genome Array. The Affymetrix Gene Chip Bovine Genome array contains 24,027 probe sets corresponding to approximately 23,000 transcripts including assemblies from ∼19,000 UniGene clusters. The arrays images were quantified using Gene Chip Operating Software (GCOS, Affymetrix).

### Gene co-expression network construction by weighted gene co-expression analysis

A component of the weighted gene WGCNA approach was initially employed to construct the network [44]. This approach has been widely employed to construct gene modules within a network based on correlations in gene expression, and the absolute Pearson correlation coefficient between gene expression levels to detect clusters of genes correlated with a trait. Networks were formed from the weighted and signed correlation matrices following the protocols of WGCNA [38].

A blockwiseModules R function was implemented using the following parameters: power 5 9, minModuleSize 5 20, deepSplit 5 0, neworkType 5 “signed”. Briefly, Pearson correlation coefficients were calculated for all pair-wise comparisons of the genes across all samples. The resulting Pearson correlation matrix was transformed into an adjacency matrix by a power function, which resulted in a weighted network. Topological overlap measure (TOM), a biologically meaningful measure of node similarity, was then calculated using a dynamic tree-cutting algorithm. Genes were hierarchically clustered using 1-TOM as the distance measure and modules were determined by choosing a height cutoff 0.995 for the resulting dendrogram. Highly similar modules were identified by clustering and merged together. The module eigengene (ME) corresponds to the first principal component of a given module. It can be considered as the most representative gene expression in a module. Module membership (MM) for each gene in each module refers to the Pearson correlation between the expression level of the gene and the ME.

### Functional annotation of modules

Annotation of network modules was performed using the Database for Annotation, Visualization and Integrated Discovery (DAVID) with the background list of all genes on the array [45]. In DAVID, an over representation of a term is defined as a modified Fisher’s exact P value with an adjustment for multiple tests using Benjamini method.

### Data analysis and visualization

Data analysis and visualization were done using R Language. All data analysis was carried out using Bioconductor packages implemented with R. Microarray expression intensities were preprocessed using protocols described in the affy package.

### Data access

All The raw microarray data have been deposited in the NCBI Gene Expression Omnibus (GEO) (http://www.ncbi.nlm.nih.gov/geo/) under accession number GSE89279.

### CONCLUSIONS

Despite numerous applications of SCNT for animal cloning, the nature of reprogramming oocyte factors and their mechanism of action remain largely unknown [4, 42, 43]. The latest transcriptome profiles of single-cell RNA-Seq reported that the human pre-implantation transcriptional organization is highly preserved, highlighting sequential order of gene activation, and genetic programming for mammalian pre-implantation development [19, 20]. In this study, we provided the first comprehensive comparisons between intra- and inter- bovine SCNT embryonic transcriptomes during preimplantation development. This study demonstrates that the inter-SCNT embryos undergo only partial or incomplete reprogramming at eight-cell stage. These results confirmed that the abnormal expression of key master pathways induced the cloned embryo developmental block. This work will contribute to a further understanding of the molecular interaction between the nuclear and the cytoplasm and provides insight into the mechanisms of cellular reprogramming.

## SUPPLEMENTARY MATERIALS FIGURES AND TABLES
















