# Supplementary material for: Coexpression analysis identifies nuclear reprogramming barriers of somatic cell nuclear transfer embryos
Source: Oncotarget. 2017 Jul 22;8(39):65847–59. doi: 10.18632/oncotarget.19504 (PMC5630377; doi:10.18632/oncotarget.19504)
Supplement: Supplementary file 1 [file oncotarget-08-65847-s001.pdf]

Coexpression analysis identifies nuclear reprogramming barriers of somatic cell nuclear transfer embryos

SUPPLEMENTARY MATERIALS

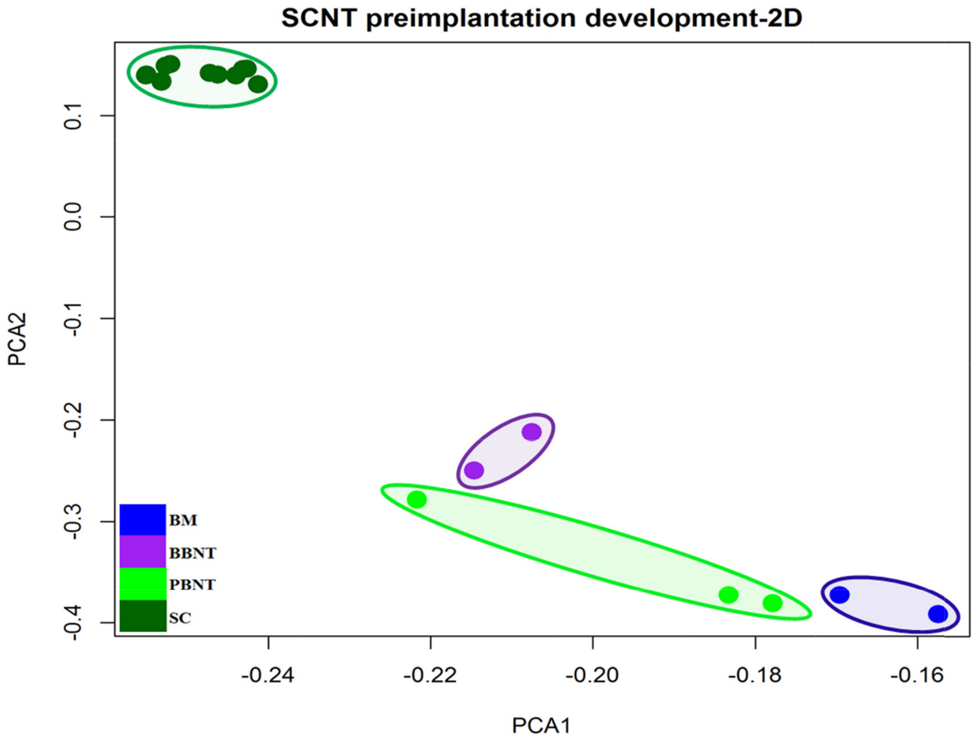

Supplementary Figure 1: The principal component analysis of BM cells (blue balls), BBNT cells (darkmagent balls), XBNT cells (green balls), and SC cells (darkgreen balls) with nearest-neighbour analysis.

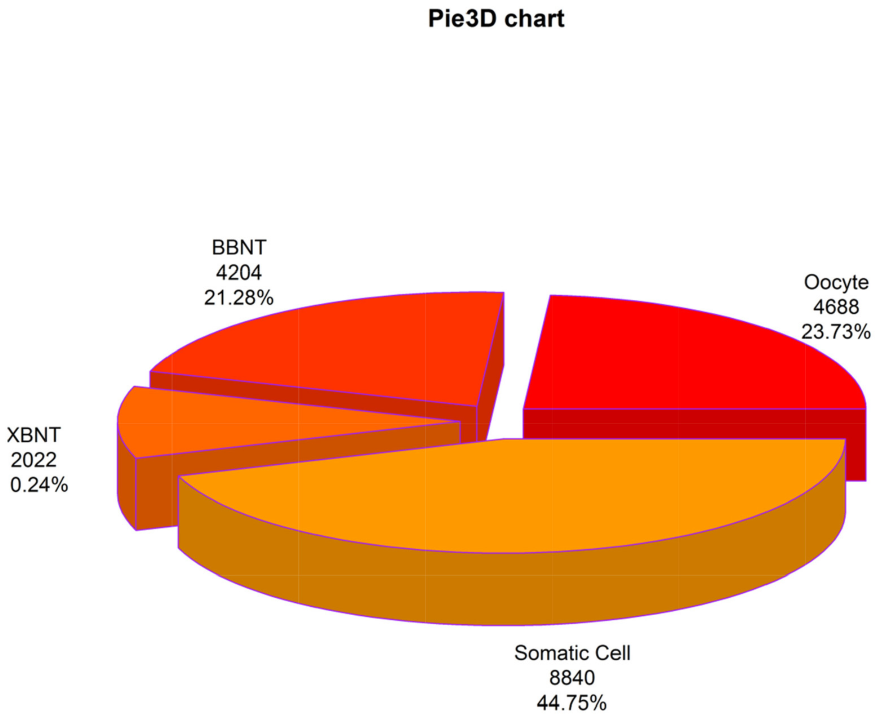

Supplementary Figure 2: The number and proportion of max expression genes for different cell types.

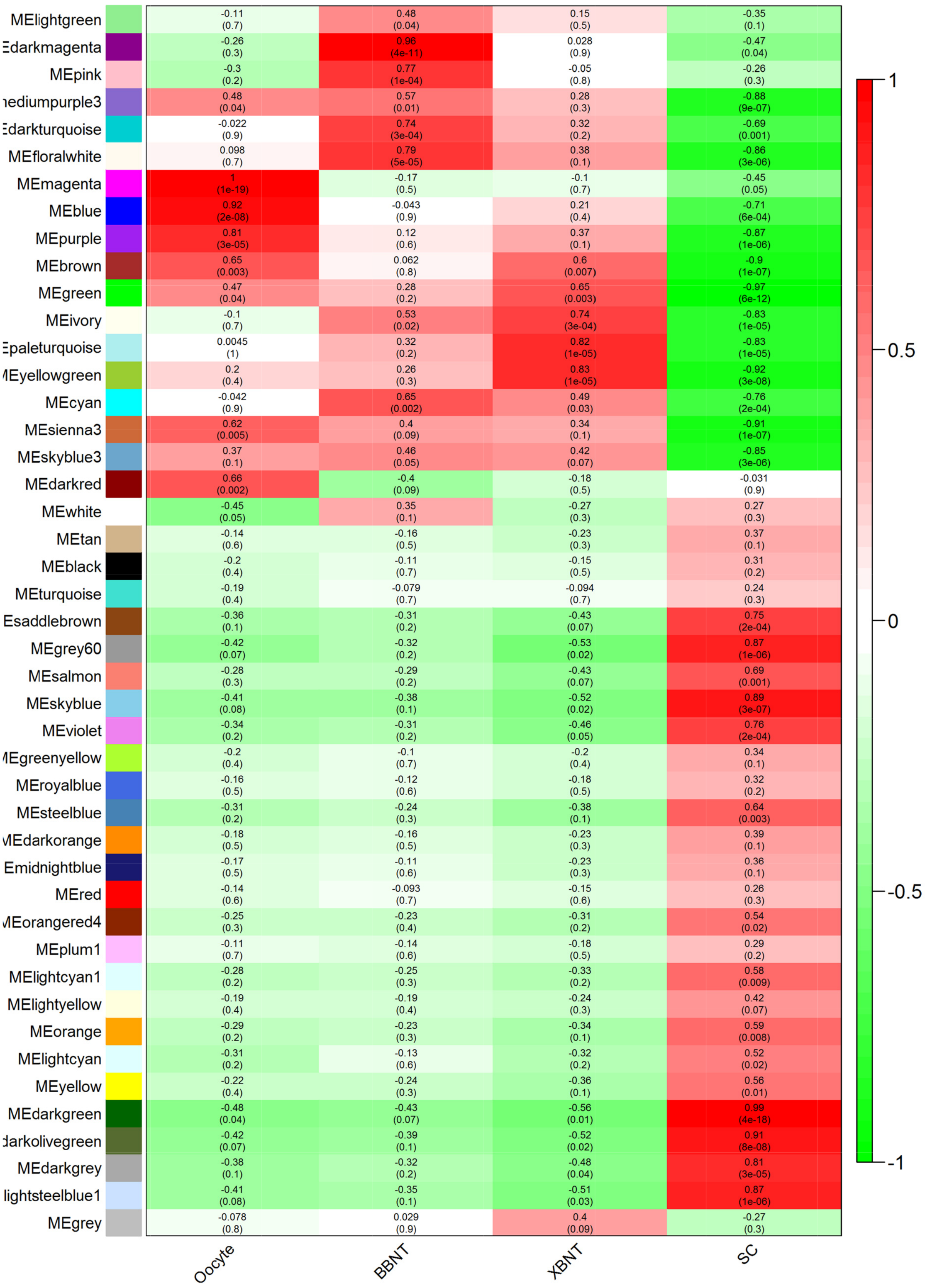

Supplementary Figure 3: The 45 colored modules identified based on gene co-expression analysis.

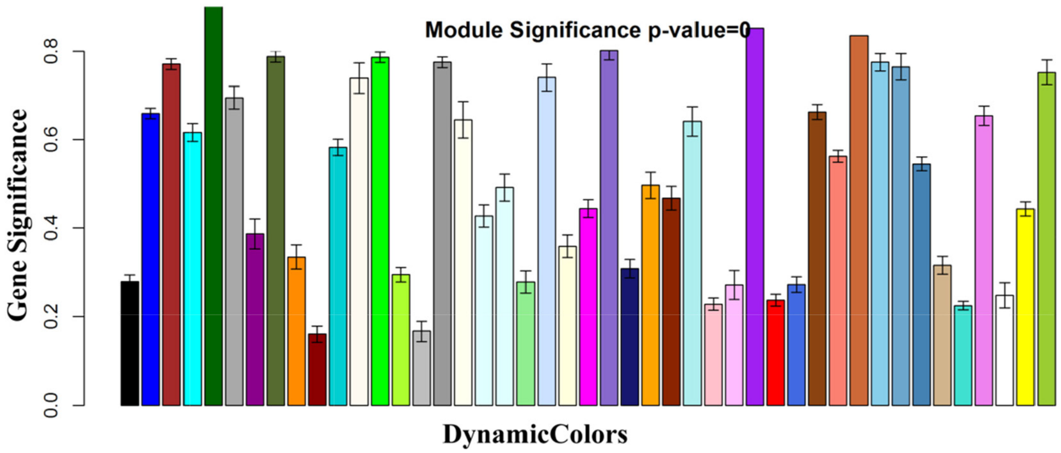

Supplementary Figure 4: The gene significance of different co-expressed models.

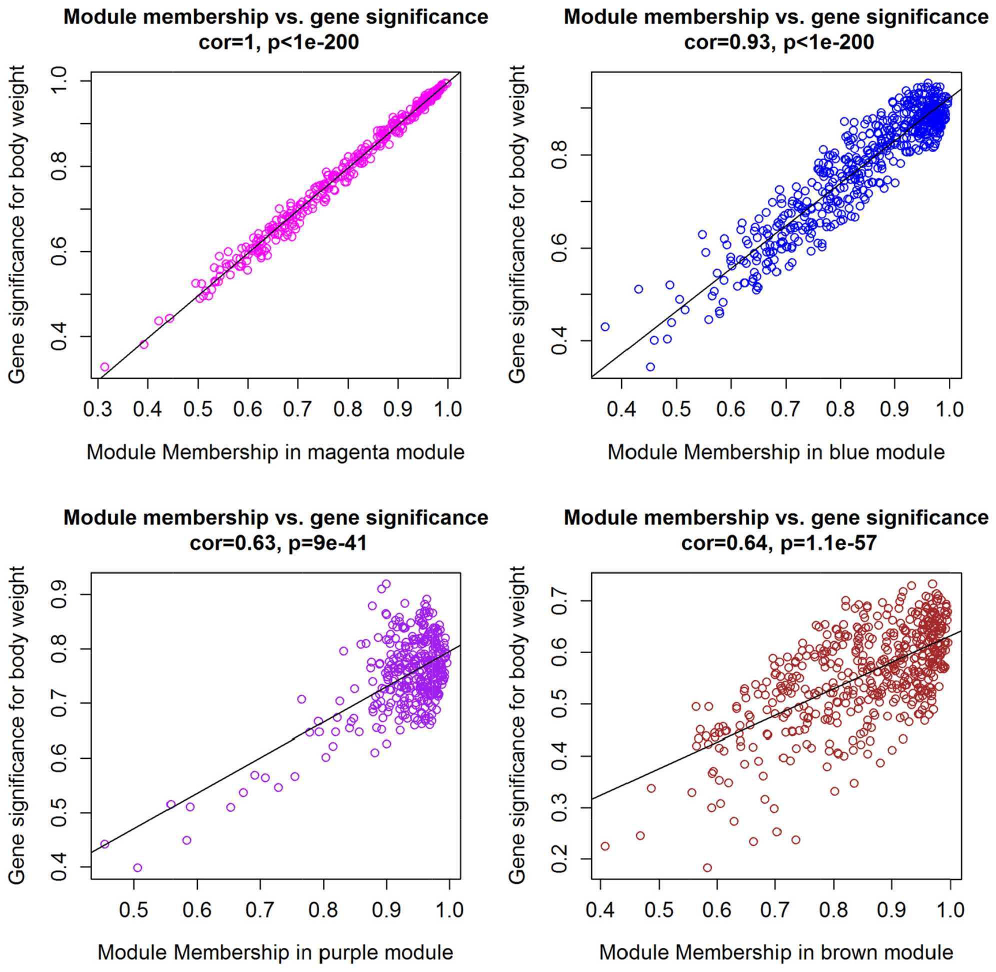

Supplementary Figure 5: Scatter relationship between module membership and gene significance for Oocytes.

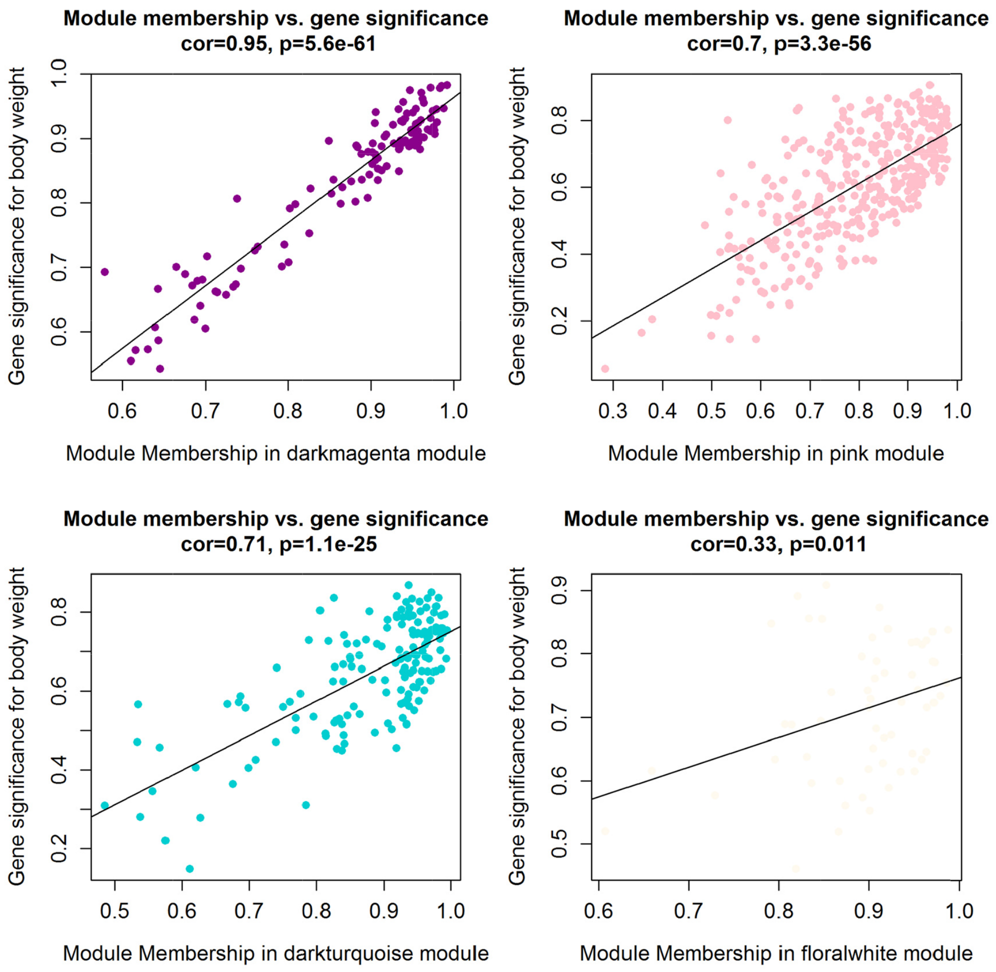

Supplementary Figure 6: Scatter relationship between module membership and gene significance for BBNT embryo.

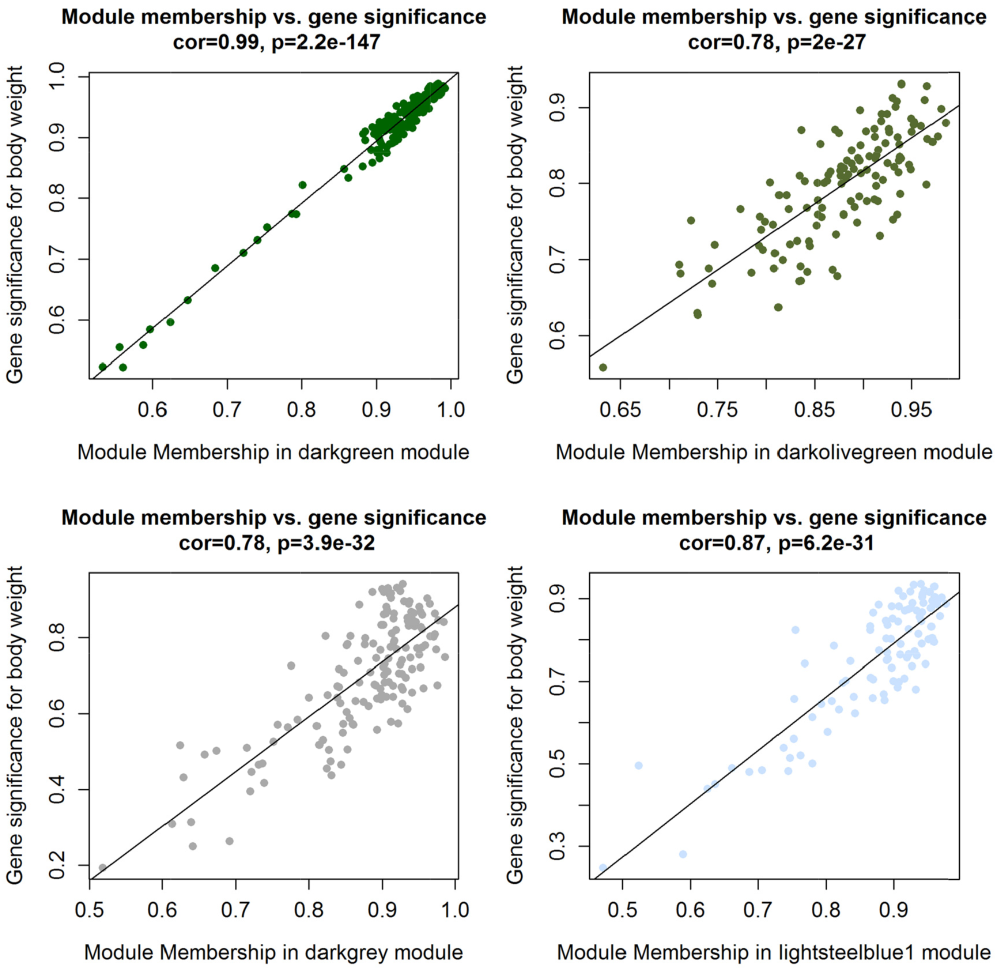

Supplementary Figure 7: Scatter relationship between module membership and gene significance for somatic cells.

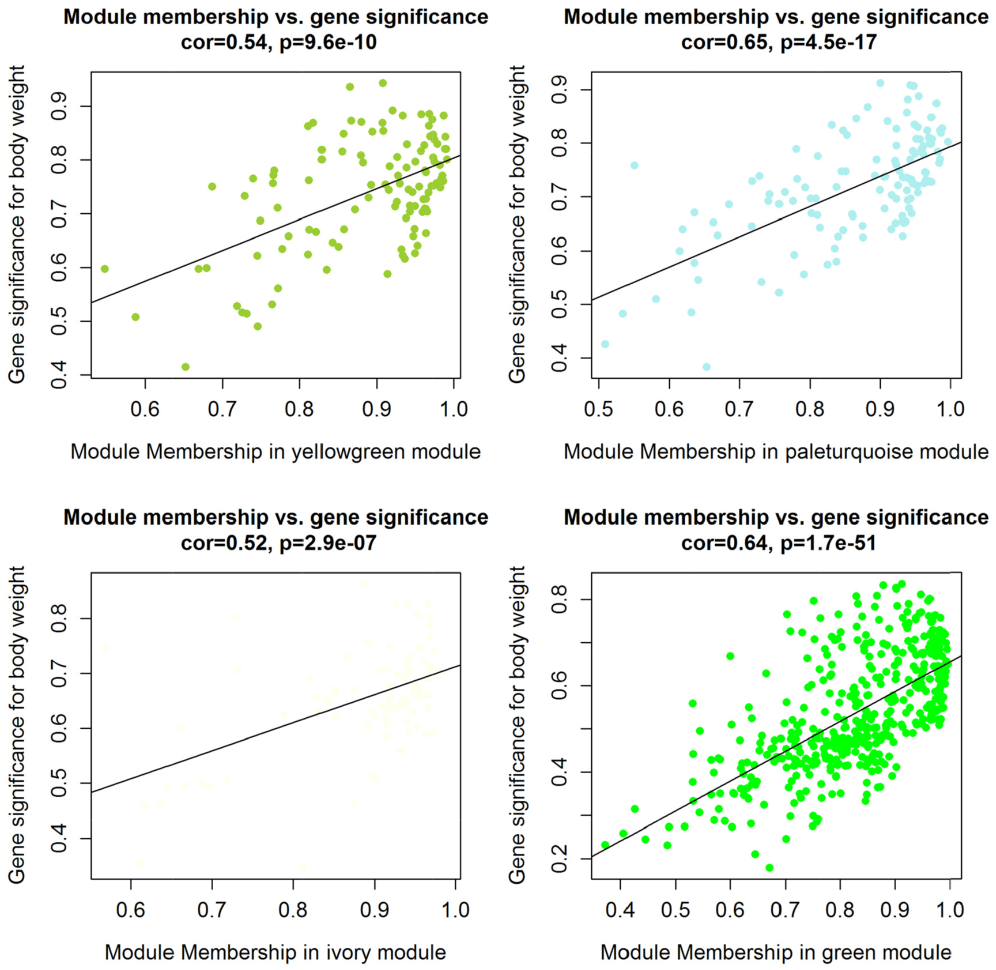

Supplementary Figure 8: Scatter relationship between module membership and gene significance for XBNT embryo.

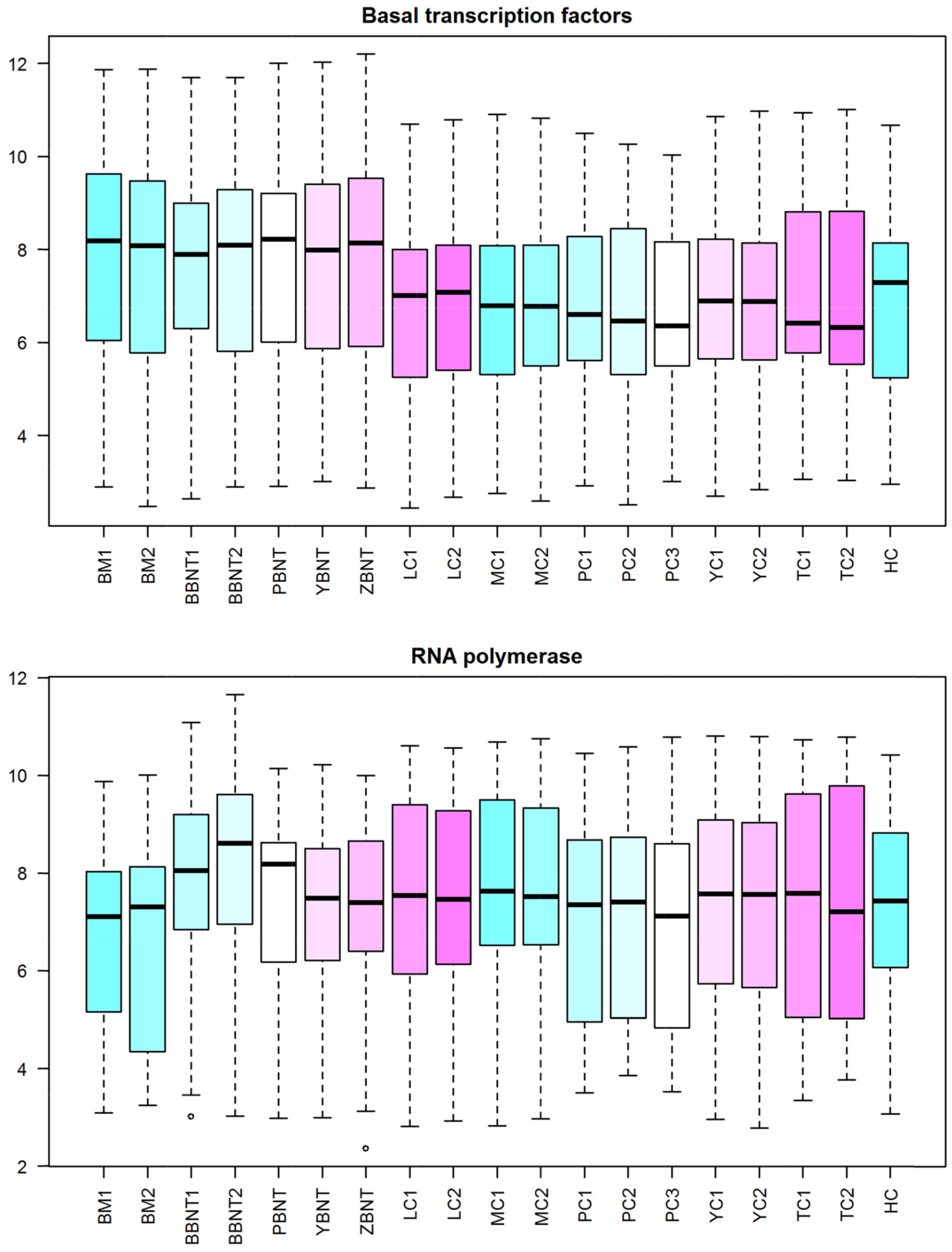

Supplementary Figure 9: The dynamic patterns of the pathways transcription regulation for different cell types.

Supplementary Table 1: The gene list of all co-expression modules

See Supplementary File 1

Supplementary Table 2: The correlation between cell type and co-expression modules by using WGCNA method

See Supplementary File 2

Supplementary Table 3: The significant between cell type and co-expression modules by using WGCNA method

See Supplementary File 3

Supplementary Table 4: The gene list of type-specific coexpression modules with Cutoff=0.74

See Supplementary File 4

Supplementary Table 5: The non-repeated GO categories of differentially expressed genes (DEGs)

See Supplementary File 5

Supplementary Table 6: The GO terms of Venn diagram shared between different cell types

See Supplementary File 6

Supplementary Table 7: The expression of key reprogramming barriers, include TFIID subunit, RNA polymerase and Mediator pathway

See Supplementary File 7
